# Genomic diversity and taxonomic marker for *Arcobacter* species

**DOI:** 10.3389/fmicb.2023.1278268

**Published:** 2023-10-10

**Authors:** Guilan Zhou, Yixin Gu, Hairui Wang, Xiaoli Chen, Xin Zhang, Zhujun Shao, Xiaomei Yan, Jianzhong Zhang, Maojun Zhang

**Affiliations:** State Key Laboratory for Infectious Disease Prevention and Control, National Institute for Communicable Disease Control and Prevention, Chinese Center for Disease Control and Prevention, Beijing, China

**Keywords:** *Arcobacter*, genome sequencing, taxonomy, ANI, *isDDH*, reliable marker gene

## Abstract

*Arcobacter* was recognized as an emerging enteropathogen and controversies regarding its classification persisted. This study aimed to reevaluate the taxonomy of *Arcobacter* utilizing the 16S rRNA gene, 23S rRNA gene, single-copy orthologous genes, as well as genomic indices such as Average Nucleotide Identity (ANI) and *in silico* DNA–DNA hybridization (*isDDH*). The taxonomy of this genus was reevaluated in this study using multiple indices with a dataset of 371 genomes comprising 34 known species and 14 potentially new species. Good discrimination could be achieved only in some species but not for the species with higher sequence similarity using the comparisons of the 16S rRNA gene and 23S rRNA gene sequences. A high-accuracy phylogenomic approach for *Arcobacter* was established using 84 single-copy orthologous genes obtained through various bioinformatics methods. One marker gene (gene711), which was found to possess the same distinguishing ability as ANI, *isDDH,* and single-copy orthologous methods, was identified as a reliable locus for inferring the phylogeny of the genus. The effective species classification was achieved by employing gene711 with a sequence similarity exceeding 96%, even for species like *A. cloacae*, *A. lanthieri*, and *A. skirrowii*, which exhibited ambiguous classification using ANI and *isDDH*. Additionally, excellent subspecies categorizing among *A. cryaerophilus* could be distinguished using gene711. In conclusion, this framework strategy had the potential advantage of developing rapid species identification, particularly for highly variable species, providing a novel insight into the behavior and characteristics of *Arcobacter*.

## Introduction

*Arcobacter* has gained increasing significance in recent years, as its members are now recognized as emerging enteropathogens and potential zoonotic agents (Ho et al., 2006). The *Arcobacter* genus belongs to the *Campylobacteraceae* family, which includes other genera: *Campylobacter*, *Helicobacter*, *Sulfurospirillum*, and others (On, 2001). Initially classified within the *Campylobacter* genus, it was in 1991 that the *Arcobacter* genus was recognized as distinct and designated as a separate genus within the *Campylobacteraceae* family (Vandamme et al., 1991). *Arcobacter* was generally described as possessing differentiated abilities from *Campylobacter*, namely the ability to grow in aerobic conditions and at temperatures between 15 and 30°C (Vandamme et al., 1992); however, this principle has been changed by the increased number of new species. Nowadays, *Arcobacter* species inhabit a wide range of ecological niches, encompassing diverse environments such as marine environments, wastewater and drinking water systems, animal feces, plants, and even oil fields, among others (Van Driessche et al., 2005; Collado and Figueras, 2011; Rathlavath et al., 2017; On et al., 2021; Pascual et al., 2023). Some *Arcobacter* species have been detected in or isolated from the stools of patients with and without diarrhea, occasionally being associated with conditions such as bacteremia, endocarditis, and peritonitis (Vandenberg et al., 2004; Ho et al., 2006; Van den Abeele et al., 2014; Isidro et al., 2020). Furthermore, it is crucial to acknowledge that the actual prevalence of *Arcobacter* species may be underestimated due to the constraints imposed by current detection and identification methods (Hanel et al., 2016). Currently, the *Arcobacter* genus consists of 34 species with validly published and accurately designated names^1^ (Pascual et al., 2023). In previous studies, the similarity of the 16S rRNA gene was considered a decisive characteristic for a taxonomic assignation at the genus level (Roth et al., 2003; Clarridge, 2004). However, misclassifications have been observed when comparing closely related species based solely on phylogenetic analysis of the 16S rRNA gene, attributed to their high sequence similarities. Debruyne et al. (2010) demonstrated that the *hsp60* gene provided higher resolution than the 16S rRNA gene in closely related species. Nonetheless, caution should be exercised when utilizing this gene alone for species-level identification within taxa characterized by high genomic diversity. Subsequently, a multilocus sequence analysis (MLSA) that relies on multiple conserved molecular markers (*atpA*, *atpD*, *dnaA*, *dnaJ*, *dnaK*, *ftsZ*, *gyrA*, *hsp60*, *radA*, *recA*, *rpoB*, *rpoD*, and *tsf*) have been investigated to differentiate species better and determine their phylogenetic relationships (Debruyne et al., 2010; Perez-Cataluna et al., 2018b). However, irrespective of the methodology employed, the identification of uncommon *Arcobacter* species remains challenging. In a taxonomy study conducted by Perez-Cataluna et al. (2018b), several approaches, including Average Nucleotide Identity (ANI), *in silico* DNA–DNA Hybridization (*isDDH*), Average Amino-acid Identity, Percentage of Conserved Proteins, and Relative Synonymous Codon Usage were employed to address this issue. The study suggested that the current *Arcobacter* genus should be divided into at least seven different genera: *Arcobacter*, *Aliarcobacter*, *Haloarcobacter*, *Pseudoarcobacter*, *Poseidonibacter*, *Malacobacter*, and Candidate ‘*Arcomarinus*’ gen. Nov (Perez-Cataluna et al., 2018b). However, On et al. (2020) revealed that the *Arcobacter* genus displayed relatively homogenous, and phylogenetic analyzes clearly distinguished this group from other *Epsilonproteobacteria* and showed that any of the measures used did not support the genomic distinction of the genera proposed by Perez-Cataluna et al. It is noteworthy that the proposal put forward by Perez-Cataluna et al. has not received approval from the International Committee on Systematics of Prokaryotes taxonomy subcommittee on *Campylobacter* or nor has it been validated in the International Journal of Systematic and Evolutionary Microbiology (On et al., 2021).

The field of prokaryotic systematics has been dramatically changed by the emergence of genome sequencing, resulting in significant advancements in various aspects, including species identification, functional characterization for taxonomic delineation, and the elucidation of phylogenetic relationships at higher taxonomic levels (Whitman, 2015). Moreover, with the advancement of detection methods, the number of *Arcobacter* strains is increasing, leading to the gradual identification of new *Arcobacter* species. Consequently, this progress poses a challenge in effectively classifying these species, thereby introducing increased difficulties in taxonomy. Incorporating genomics into taxonomy appears to be a promising development, enhancing credibility by offering reproducible, reliable, and highly informative methods to infer phylogenetic relationships among prokaryotes while avoiding unreliable approaches and subjective, difficult-to-replicate data. Within this modern taxonomy context, the objective of this study was to reassess the taxonomy of both known and newly identified *Arcobacter* species by using 16S rRNA gene, 23S rRNA gene, the whole genome sequences, and the derived genomic analysis, providing valuable insights into the taxonomic investigation of *Arcobacter*. We also evaluated the efficacy of various genome-based phylogenetic tools in discriminating between different *Arcobacter* species.

## Materials and methods

### Bacterial strains

In this study, 371 *Arcobacter* genomes were used, out of which 172 were obtained from strains sequenced by our laboratory or collaborating institutions. The isolation, cultivation, genomic DNA extraction, and sequencing of these strains were described in previous publications (Wang et al., 2021; Ma et al., 2022; Zhou et al., 2022). Furthermore, genomes of *Arcobacter* identified at the species level were investigated, 172 of which were obtained in our earlier studies (70 *A. butzleri*, 81 *A. cryaerophilus*, 19 *A. skirrowii*, and 2 *A. lacus*), and the others from the public databases. checkM software (Parks et al., 2015) was used to assess genomic contamination and completeness, resulting in contamination <4.67% (CNAS04, CNAC065) and completeness >96.34% (CNAB027). The 371 genomes were annotated with a local installation of Prokka v1.14.6 (Seemann, 2014) with the prediction tools Prodigal v2.6.3 (Hyatt et al., 2010) and ARAGORN v1.2.41 (Laslett and Canback, 2004). The prediction tool barrnap v0.9^2^ included in Prokka v1.14.6 was used to annotate rRNA genes. The characteristics of each genome (i.e., N50, number of contigs, G + C content) were obtained using in-house scripts.

### Downloading of publicly available genomes

All 34 valid species included in the *Arcobacter* genus have been studied. They were represented by 199 genomes and 17 potentially new species genomes (Supplementary Table S1). All genome sequences identified as *Arcobacter* were downloaded from the National Center for Biotechnology Information (NCBI) and Bacterial and Viral Bioinformatics Resource Center (BV-BRC) public database on January 2023. All publicly available assemblies were subjected to quality control by Quast software (Gurevich et al., 2013). Firstly, genomic sequences identified as “poor” were excluded from the analysis based on the sequencing quality. Secondly, genomes that did not meet the criteria for genome size and GC content were filtered out according to the genomic characteristics of *Arcobacter*. Additionally, only genomes with a scaffold count of less than 200 were included to ensure the reliability of the analysis results. Finally, the obtained genomes underwent species identification using the GTDB v2.3.2 software (Chaumeil et al., 2019), and only the genomes identified as *Arcobacter* were included in the analysis. A total of 199 *Arcobacter* genomes were included in the study, comprising 34 named *Arcobacter* species and 14 unclassified *Arcobacter* species, as shown in Supplementary Table S1.

### Analysis of ribosomal genes

The 16S rRNA gene and 23S rRNA gene sequences were extracted from the genome assemblies using barrnap v0.9, producing a gff file of rRNA gene locations in the genome assemblies. The gff files were combined with the bedtools (Quinlan and Hall, 2010), fastaFromBed, to extract the 16S rRNA and 23S rRNA gene sequences from the genome assemblies. Genes sequences were aligned using MAFFT v7.490 software (Katoh and Standley, 2013). The genomes containing the complete 16S rRNA gene and 23S rRNA gene were selected, and the corresponding sequences were extracted and aligned to construct a Neighbor-Joining (NJ) phylogenetic tree with a bootstrap value of 1,000. Additionally, pairwise sequence comparisons were performed using MAFFT v7.490 software (Katoh and Standley, 2013) to determine sequence alignments and assess the similarity between pairs of sequences.

### Analysis of ANI and *isDDH*

Pairwise ANI values were calculated for all genomes using pyani v0.2 software (module ANIb), accessible at https://github.com/widdowquinn/pyani. The Genome-to-Genome Distance Calculator (GGDC) web service was used to report *isDDH* for the accurate delineation of prokaryotic subspecies and to calculate differences in G + C genomic content.^3^ Analysis was performed using “Formula 2,” as recommended by the GGDC authors, which allows for *isDDH* estimation independent of genome lengths, making it suitable for incomplete genomes. A matrix with ANI values across all genomes was visualized using the pheatmap package, and an in-house script was used to generate a clustering dendrogram based on the ANI matrix.

### Identification of single-copy orthologous genes and marker gene

The OrthoFinder v2.5.4 software (Emms and Kelly, 2019) was employed to perform a homology analysis on the 371 *Arcobacter* genomes, identifying single-copy orthologous genes. The software parameters used were -S blast, −M msa, −T raxml. The EasyTree.py script^4^ was used to extract all single-copy orthologous genes from each genome. The genes were aligned using the MAFFT v7.490 software, and an ML tree (data not shown) was constructed by concatenating and coalescing these genes using the raxmlHPC v8.2.12 software (Stamatakis, 2014) and MEGA 7 (Kumar et al., 2016) software, with a bootstrap value of 1,000. The resulting tree was annotated using the table2itol package and visualized in iTOL.^5^

## Results

### Genomic characteristics of the *Arcobacter*

A total of 371 high-quality sequenced and assembled genomes of *Arcobacter* were obtained through genome quality control, and a comprehensive analysis was conducted on 371 genomes. All 34 species currently included in the *Arcobacter* and 14 candidate species have been investigated in the present study. The scaffolds obtained and the N50 values complied with the proposed minimal standards for using genomes in taxonomic studies (Chun et al., 2018). Genome assemblies had 1 to 166 contigs. The genome sizes and GC contents displayed significant variations across different *Arcobacter* species. The genome size ranged from 1.68 Mb for *A. skirrowii* CNAS13 to 3.57 Mb for *A. lekithochrous* CP054052. The genome size of *A. skirrowii* was generally smaller than that of other *Arcobacter* species. In comparison, the genome size of *A. lekitochrous* was generally larger than that of other *Arcobacter* species. The G + C content ranged from 26.08% in *A. molluscorum* NXFY00000000 to 31.00% in *Arcobacter* spp. JAIFNA000000000, as shown in Supplementary Table S1.

### Phylogenetic of ribosomal genes

**Figure 1:**
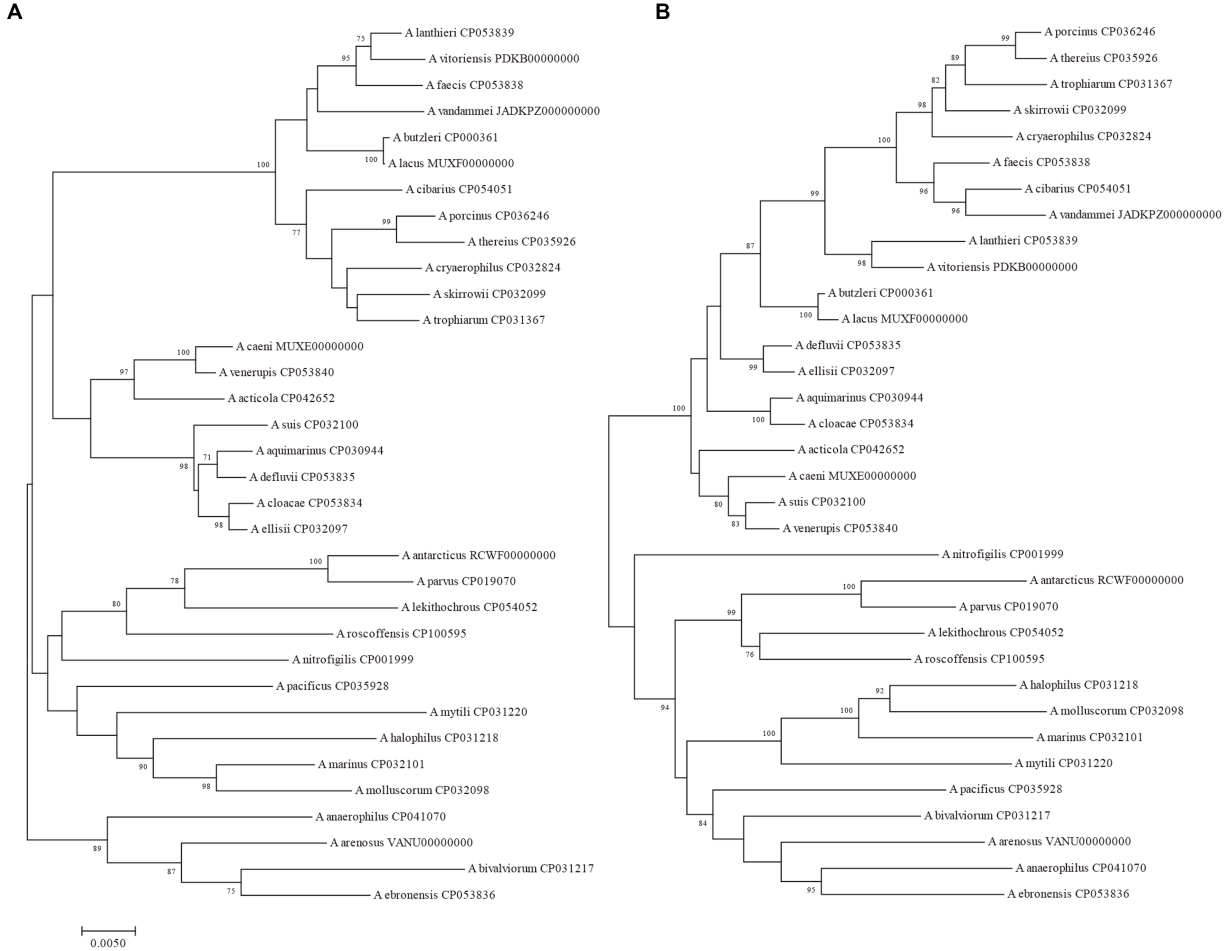
The NJ tree was constructed based on the 16S rRNA gene and 23S rRNA gene sequences of 34 *Arcobacter* species type strains, with a bootstrap value of 1,000. **(A)** The phylogenetic tree was constructed using the 16S rRNA gene, and **(B)** was constructed using the 23S rRNA gene. Bar indicated 5 substitutions per 1,000 bp.

**Figure 2:**
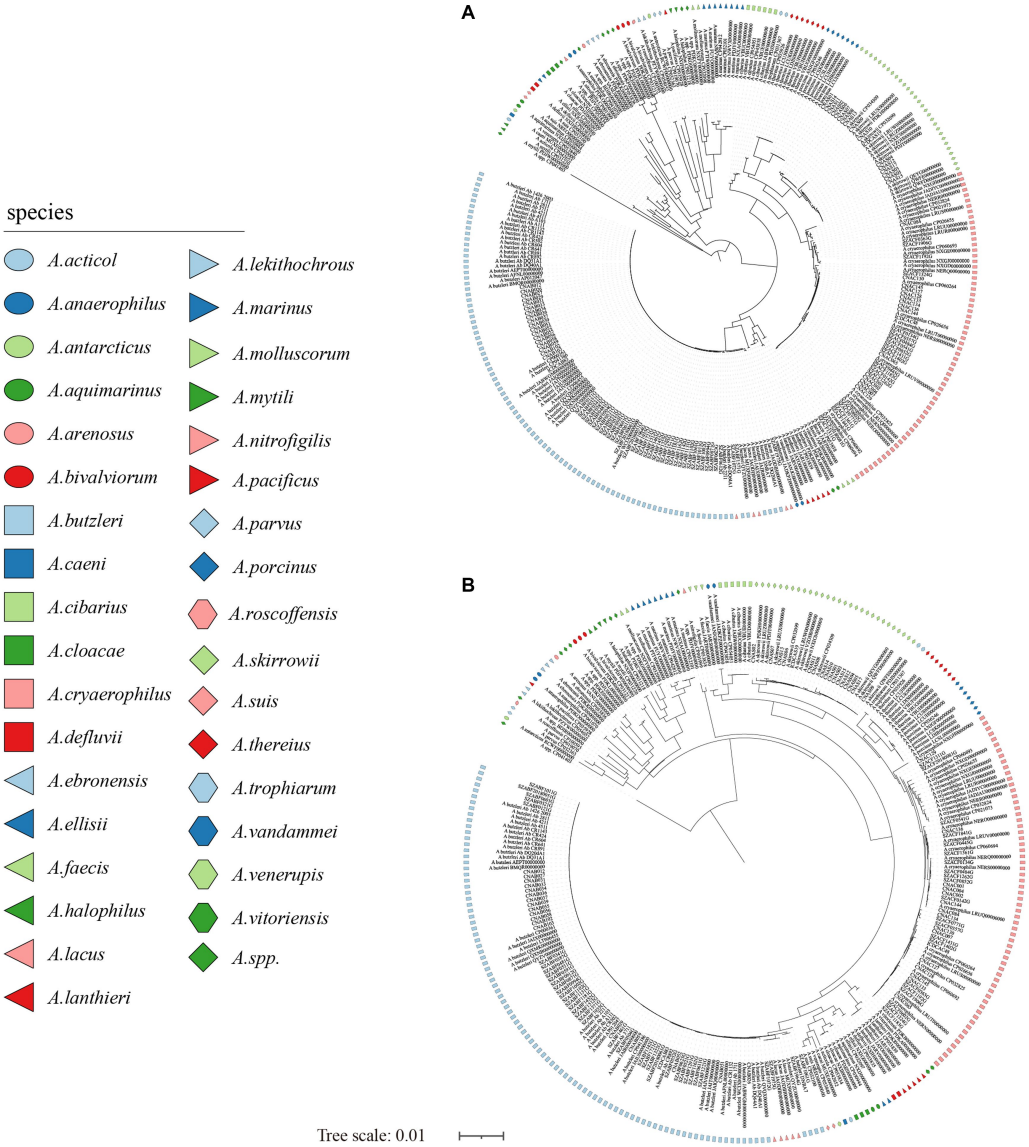
The NJ tree was constructed based on the 16S rRNA gene and 23S rRNA gene sequences of 281 *Arcobacter* genomes, with a bootstrap value of 1,000. **(A)** The phylogenetic tree was constructed using the 16S rRNA gene, and **(B)** was constructed using the 23S rRNA gene. Different colors or shapes indicated different *Arcobacter* species. Bar indicated 1 substitution per 100 bp.

The size of the 16S rRNA gene in 34 type strains of *Arcobacter* species ranged from 1,512 to 1,516 bp, with sequence similarities ranging from 91.97% (between *A. cryaerophilus* and *A. bivalviorum*) to 99.93% (between *A. butzleri* and *A. lacus*). Similarly, the size of the 23S rRNA gene varied from 2,873 to 3,026 bp, with sequence similarities ranging from 86.72% (between *A. vandammei* and *A. pacificus*) to 99.72% (between *A. butzleri* and *A. lacus*). Detailed results can be found in Tables 1, 2 and Supplementary Table S2. The phylogenetic trees constructed based on the 16S rRNA gene and 23S rRNA gene of the type strains were presented in Figure 1. It was noteworthy that there were certain variations observed in the phylogenetic trees constructed using different sequence datasets. Of the 371 *Arcobacter* genomes analyzed, 281 were selected for analysis due to the near-full length of the 16S rRNA gene and 23S rRNA gene. The size of the 16S rRNA gene ranged from 1,306 to 1,517 bp, almost all of which were around 1,514 bp, except VBUD00000000, VBUC00000000, NXGJ00000000, SZACF0142G, SZACF1311G, and SZACF1324G. Similarly, the size of the 23S rRNA gene ranged from 2,607 to 3,030 bp, most of which were around 2,907 bp. The similarities in the 16S rRNA gene sequences among different *Arcobacter* species (all the 34 species currently included in the genus and the 14 new candidate species) showed a wide range of values (Table 2; Supplementary Table S2). Similarities ranged from 89.10% (between *A. anaerophilus_*CP041070 and *A*. spp_CP041403) to 100% (between *A. butzleri* and *A. lacus*). Notably, the similarity of the 16S rRNA gene between some *Arcobacter* species reached or even exceeded the similarity within species, such as *A. cloacae* and *A. ellisii*, *A. cryaerophilus* and *A. skirrowii*, *A. lacus* and *A. butzleri* and others. The differences in 23S rRNA gene sequences among different *Arcobacter* species were greater compared to the 16S rRNA gene sequences, with sequence similarities ranging from 83.60% (between *A. vandammei* and *A. marinus*) to 99.76% (between *A. butzleri* and *A. lacus*). However, the similarity of 23S rRNA gene sequences among some species still exceeded the similarity within species such as *A. cryaerophilus* and *A. skirrowii*, *A. lacus* and *A. butzleri*. Figure 2 and Supplementary Figure S1 illustrate the phylogenetic relationships of the 16S rRNA gene and 23S rRNA gene among the presently described species. Although these two phylogenetic trees showed high topological similarity, neither of them effectively distinguished species within the *Arcobacter* genus, as evidenced by the inability to differentiate between *A. butzleri* and *A. lacus.* For most species of *Arcobacter*, phylogenetic trees based on 16S rRNA and 23S rRNA genes have better resolution.

**Table 1:** 16S rRNA gene and 23S rRNA gene sizes and the start and end positions of gene711 in the genome of 34 *Arcobacter* species type strains.

Species	GenBank Accession (Type Strain)	16S rRNA	23S rRNA	gene711	gene711_Start	gene711_End	Note*
*Arcobacter acticola*	CP042652	1,514	2,949	597	2,885,771	2,886,367	
*Arcobacter anaerophilus*	CP041070	1,514	2,878	618	2,913,613	2,914,230	
*Arcobacter antarcticus*	RCWF00000000	1,514	2,909	585	112,316	112,900	fasta56
*Arcobacter aquimarinus*	CP030944	1,515	2,908	585	2,431,557	2,432,141	
*Arcobacter arenosus*	VANU00000000	1,515	2,873	609	378,208	378,816	fasta12
*Arcobacter bivalviorum*	CP031217	1,515	2,905	654	2,582,303	2,582,956	
*Arcobacter butzleri*	CP000361	1,514	2,907	591	2,262,773	2,263,363	
*Arcobacter caeni*	MUXE00000000	1,514	2,906	618	15,779	16,396	fasta57
*Arcobacter cibarius*	CP054051	1,515	2,908	585	1,968,875	1,969,459	
*Arcobacter cloacae*	CP053834	1,515	2,908	588	2,533,454	2,534,041	
*Arcobacter cryaerophilus*	CP032824	1,515	2,907	600	1,897,718	1,898,317	
*Arcobacter defluvii*	CP053835	1,515	2,906	588	2,904,720	2,905,307	
*Arcobacter ebronensis*	CP053836	1,515	2,879	615	3,035,147	3,035,761	
*Arcobacter ellisii*	CP032097	1,515	2,906	600	2,702,365	2,702,964	
*Arcobacter faecis*	CP053838	1,515	2,907	588	1,991,920	1,992,507	
*Arcobacter halophilus*	CP031218	1,514	2,908	576	2,741,754	2,742,329	
*Arcobacter lacus*	MUXF00000000	1,514	2,907	603	98,969	99,571	fasta16
*Arcobacter lanthieri*	CP053839	1,515	2,907	603	122,801	123,403	
*Arcobacter lekithochrous*	CP054052	1,514	2,908	588	3,484,870	3,485,457	
*Arcobacter marinus*	CP032101	1,512	2,915	576	2,842,957	2,843,532	
*Arcobacter molluscorum*	CP032098	1,514	2,908	576	2,724,302	2,724,877	
*Arcobacter mytili*	CP031220	1,514	2,908	573	2,795,077	2,795,649	
*Arcobacter nitrofigilis*	CP001999	1,514	2,911	558	3,065,193	3,065,750	
*Arcobacter pacificus*	CP035928	1,514	2,921	597	2,556,007	2,556,603	
*Arcobacter parvus*	CP019070	1,514	2,909	585	2,800,450	2,801,034	
*Arcobacter porcinus*	CP036246	1,515	2,907	615	90,336	90,950	
*Arcobacter skirrowii*	CP032099	1,514	2,909	618	126,189	126,806	
*Arcobacter suis*	CP032100	1,515	2,906	615	2,530,252	2,530,866	
*Arcobacter thereius*	CP035926	1,515	2,907	615	91,036	91,650	
*Arcobacter trophiarum*	CP031367	1,515	2,908	597	103,102	103,698	
*Arcobacter vandammei*	JADKPZ000000000	1,514	3,026	588	58,759	59,346	fasta23
*Arcobacter venerupis*	CP053840	1,514	2,906	609	3,051,151	3,051,759	
*Arcobacter vitoriensis*	PDKB00000000	1,515	2,907	603	71,009	71,611	fasta5
*Arcobacter roscoffensis*	CP100595	1,514	2,908	588	3,076,989	3,077,576	

*Arcobacter antarcticus*, *Arcobacter arenosus*, *Arcobacter caeni*, *Arcobacter lacus*, *Arcobacter vandammei*, and *Arcobacter vitoriensis* are draft genomes, and the start and end positions of gene711 are scaffold56, scaffold12, scaffold57, scaffold16, scaffold23, and scaffold5, respectively.

**Table 2:** Intraspecies and interspecies similarity of 16S rRNA gene, 23S rRNA gene, ANI, and gene711 of *Arcobacter*.

	16S rRNA gene in intraspecies	16S rRNA gene in interspecies (Closest related)	23S rRNA gene in intraspecies	23S rRNA gene in interspecies (Closest related)	ANI in intraspecies	ANI in interspecies (Closest related)	gene711 in intraspecies	gene711 in interspecies (Closest related)
*A. acticola*	100%	*A. venerupis* (98.40%)	100%	*A. caeni* (96.91%)	100.00%	*A. caeni* (83.06%)	100.00%	*A. cloacae* (77.22%)
*A. anaerophilus*	100%	*A. ebronensis* (96.60%)	100%	*A. ebronensis* (96.25%)	100.00%	*A. ebronensis* (79.13%)	100.00%	*A. ebronensis* (69.26%)
*A. antarcticus*	100%	*A. parvus* (98.50%)	100%	*A. parvus* (97.42%)	100.00%	*A. parvus* (84.04%)	100.00%	*A*. spp*_*CAJWWD000000000 (77.44%)
*A. aquimarinus*	100%	*A. defluvii* (99.41%)	100%	*A. cloacae* (99.42%)	97.05%	*A. cloacae* (93.82%)	100.00%	*A. cloacae* (93.88%)
*A. arenosus*	100%	*A*. spp_PDKF00000000 (96.70%)	100%	*A. ebronensis* (95.59%)	100.00%	*A. bivalviorum* (78.01%)	100.00%	*A. ebronensis* (60.89%)
*A. bivalviorum*	99.60%	*A*. spp_PDJU00000000 (99.27%)	99.93%	*A*. spp*_*PDKF00000000 (97.04%)	96.52%	*A*. spp*_*PDJU00000000 (85.66%)	97.71%	*A*. spp_PDJU00000000 (84.40%)
*A. butzleri*	99.93%	*A. lacus* (100%)	99.73%	*A. lacus* (99.76%)	96.75%	*A. lacus* (94.60%)	96.62%	*A. lacus* (84.41%)
*A. caeni*	100%	*A. venerupis* (99.47%)	100%	*A. suis* (99.04%)	100.00%	*A. suis* (87.86%)	100.00%	*A. suis* (86.41%)
*A. cibarius*	99.92%	*A. cryaerophilus* (99.01%)	100%	*A. faecis* (98.35%)	98.85%	*A. faecis* (84.79%)	98.97%	*A. vandammei* (84.69%)
*A. cloacae*	99.80%	*A. ellisii* (99.80%)	99.90%	*A. aquimarinus* (99.42%)	95.61%	*A. aquimarinus* (93.82%)	96.43%	*A. aquimarinus* (93.88%)
*A. cryaerophilus*	98.95%	*A. skirrowii* (99.51%)	93.05%	*A. skirrowii* (99.11%)	92.32%	*A. trophiarum* (85.78%)	92.33%	*A. trophiarum* (88.83%)
*A. cryaerophilus-I*	99.41%	*A. cryaerophilus-II* (100%)	93.05%	*A. cryaerophilus-2* (99.76%)	96.00%	*A. cryaerophilus-II* (96.26%)	95.33%	*A. cryaerophilus-II* (96.50%)
*A. cryaerophilus-II*	99.34%	*A. cryaerophilus-I* (100%)	99.48%	*A. cryaerophilus-3* (99.90%)	96.33%	*A. cryaerophilus-I* (96.26%)	98.17%	*A. cryaerophilus-I* (96.50%)
*A. cryaerophilus-III*	100%	*A. cryaerophilus-II* (99.93%)	100%	*A. cryaerophilus-2* (99.90%)	100.00%	*A. cryaerophilus-IV* (94.26%)	100.00%	*A. cryaerophilus-IV* (95.83%)
*A. cryaerophilus-IV*	100%	*A. cryaerophilus-III* (99.54%)	100%	*A. cryaerophilus-3* (99.31%)	97.78%	*A. cryaerophilus-III* (94.34%)	99.00%	*A. cryaerophilus-III* (95.83%)
*A. defluvii*	100%	*A. cloacae/A.aquimarinus* (99.41%)	100%	*A. ellisii* (99.38%)	100.00%	*A. aquimarinus* (84.46%)	100.00%	*A. cloacae* (85.67%)
*A. ebronensis*	99.47%	*A. bivalviorum* (96.70%)	99.50%	*A. anaerophilus* (96.25%)	96.10%	*A. anaerophilus* (79.13%)	96.75%	*A. anaerophilus* (69.26%)
*A. ellisii*	100%	*A. cloacae* (99.80%)	100%	*A. defluvii* (99.38%)	96.42%	*A. defluvii* (87.58%)	98.00%	*A. defluvii* (85.67%)
*A. faecis*	100%	*A. lanthieri* (99.14%)	100%	*A. cibarius* (98.35%)	99.80%	*A. vandammei* (85.20%)	100.00%	*A. vandammei* (85.03%)
*A. halophilus*	100%	*A. marinus* (96.63%)	100%	*A. molluscorum* (96.87%)	99.97%	*A. marinus* (86.55%)	100.00%	*A. marinus* (86.11%)
*A. lacus*	99.93%	*A. butzleri* (100%)	99.97%	*A. butzleri* (99.76%)	100.00%	*A. butzleri* (94.56%)	99.50%	*A. butzleri* (84.41%)
*A. lanthieri*	99.80%	*A. vitoriensis* (99.27%)	99.62%	*A. vitoriensis* (98.87%)	95.67%	*A. vitoriensis* (87.69%)	96.19%	*A. vitoriensis* (87.40%)
*A. lekithochrous*	100%	*A. roscoffensis* (96.51%)	100%	*A. roscoffensis* (96.60%)	98.33%	*A. roscoffensis* (79.24%)	98.64%	*A. roscoffensis* (74.66%)
*A. marinus*	99.60%	*A. molluscorum* (97.69%)	93.81%	*A. halophilus* (96.33%)	95.26%	*A. halophilus* (86.48%)	94.79%	*A. halophilus* (86.11%)
*A. molluscorum*	100%	*A*. spp_PDKC00000000 (98.55%)	100%	*A*. spp_PDKG00000000 (98.56%)	100.00%	*A*. spp*_*PDKG00000000 (90.23%)	100.00%	*A*. spp*_*PDKG00000000 (88.54%)
*A. mytili*	99.93%	*A*. spp_PDKG00000000 (95.24%)	99.69%	*A. halophilus* (94.98%)	98.97%	*A. halophilus* (80.80%)	100.00%	*A. molluscorum* (74.44%)
*A. nitrofigilis*	100%	*A*. spp_PDJV00000000 (99.21%)	100%	*A*. spp*_*PDJV00000000 (99.28%)	100.00%	*A*. spp_PDJV00000000 (91.83%)	100.00%	*A*. spp*_*PDJV00000000 (86.38%)
*A. pacificus*	100%	*A. roscoffensis* (96.30%)	100%	*A. arenosus* (93.37%)	99.86%	*A. cloacae* (78.88%)	100.00%	*A. parvus* (72.26%)
*A. parvus*	99.87%	*A. antarcticus* (98.55%)	99.38%	*A. antarcticus* (97.42%)	98.09%	*A. antarcticus* (84.27%)	99.49%	*A*. spp*_*CAJWWD000000000/*A.spp_*NYWO00000000 (78.46%)
*A. porcinus*	100%	*A. thereius* (99.01%)	99.90%	*A. thereius* (99.42%)	98.22%	*A. thereius* (93.51%)	99.84%	*A. thereius* (92.52%)
*A. skirrowii*	99.14%	*A. cryaerophilus* (99.51%)	92.85%	*A. cryaerophilus* (99.11%)	94.83%	*A. cryaerophilus* (94.83%)	96.12%	*A. thereius* (78.69%)
*A. suis*	99.93%	*A. cloacae* (99.08%)	100%	*A. venerupis* (99.35%)	99.92%	*A. caeni* (87.94%)	100.00%	*A. caeni* (86.41%)
*A. thereius*	100%	*A. porcinus* (99.01%)	100%	*A. porcinus* (99.42%)	98.63%	*A. porcinus* (93.49%)	99.35%	*A. porcinus* (92.52%)
*A. trophiarum*	99.93%	*A. cryaerophilus* (98.94%)	100%	*A. porcinus* (98.62%)	99.74%	*A. cryaerophilus* (85.82%)	100.00%	*A. cryaerophilus* (88.83%)
*A. vandammei*	100%	*A. lanthieri* (98.42%)	100%	*A. skirrowii* (96.80%)	100.00%	*A. faecis* (85.25%)	100.00%	*A. faecis* (85.03%)
*A. venerupis*	100%	*A. caeni* (99.47%)	100%	*A. suis* (99.35%)	100.00%	*A. suis* (86.65%)	100.00%	*A. caeni* (79.94%)
*A. vitoriensis*	99.93%	*A. lanthieri* (99.27%)	100%	*A. lanthieri* (98.87%)	97.31%	*A. lanthieri* (87.19%)	98.84%	*A. lanthieri* (87.40%)
*Arcobacter roscoffensis*	100%	*A. pacificus* (96.30%)	100%	*A. lekithochrous* (96.60%)	100.00%	*A. lekithochrous* (79.24%)	100.00%	*A. lekithochrous* (74.66%)

The highlighted content in the table indicates species with ambiguous classifications when employing various indices for species identification.

### Species classification and genetic population

**Figure 3:**
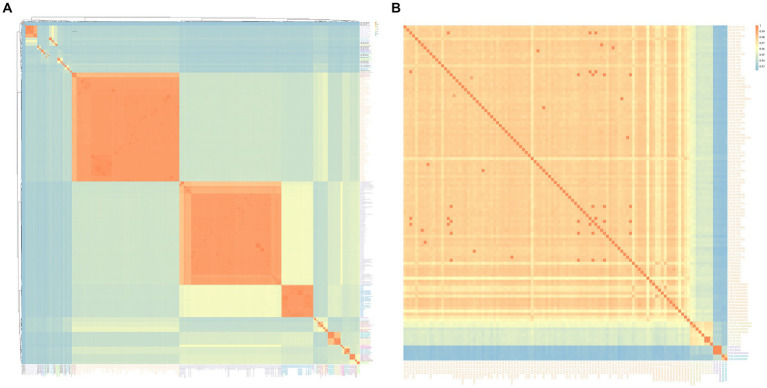
*Arcobacter* ANIb heatmap using the pheatmap package. **(A)** was the ANIb heatmap of 34 known *Arcobacter* species and 14 unknown *Arcobacter* species, and **(B)** was the heatmap of *A. cryaerophilus*. The depth of the color indicated the size of the ANI value, which increased sequentially from blue to orange.

The results of the ANI and the *isDDH* calculations among the studied genomes were given in Table 2, Supplementary Table S3, and Figure 3. Significant differences in ANI were observed among different species of *Arcobacter*. The ANI values among some strains within *A. cloacae*, *A. lanthieri*, *A. marinus*, *A. skirrowii*, and *A. cryaerophilus* species were < 96%, and the *isDDH* values were < 70%. Among them, the most significant differences in ANI and *isDDH* were observed between subspecies of *A. cryaerophilus*, with ANI and *isDDH* values of 92.32 and 48.10%, respectively. However, the ANI or *isDDH* values within the species were significantly higher than those with the closest related species. In addition to the known species of *Arcobacter*, 17 genomes potentially represented 14 new species that were identified. The ANI values between these new species and the known genomes of *Arcobacter* exhibited significant differences. The ANI and *isDDH* values compared to known *Arcobacter* species were below 96 and 70%, respectively, which were the cut-off values proposed for delineating new species. Only the ANI between *A*. spp._PDJV00000000 and *A. nitrofigilis*_CP001999 > 90%, while for the remaining genomes <90%.

### Phylogenetic reconstruction using the marker gene

**Figure 4:**
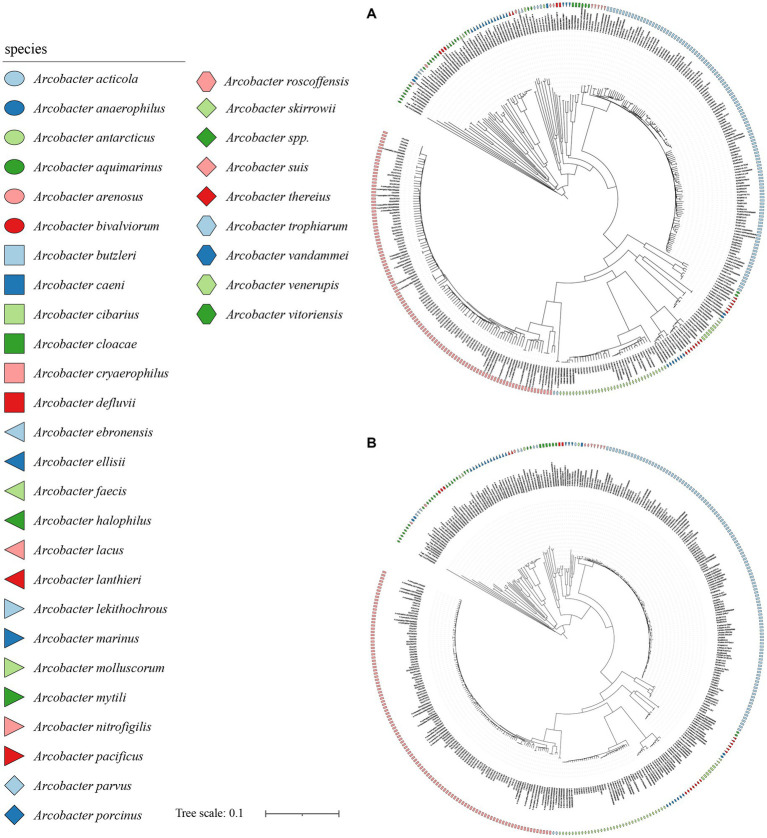
The NJ tree was constructed based on the 84 single-copy homologous genes, with a bootstrap value of 1,000. **(A)** was the phylogenetic tree constructed using nucleotide sequence, and **(B)** was the phylogenetic tree constructed using amino acid sequence. Different colors or shapes indicated different *Arcobacter* species. Bar indicated 1 substitution per 10 bp.

**Figure 5:**
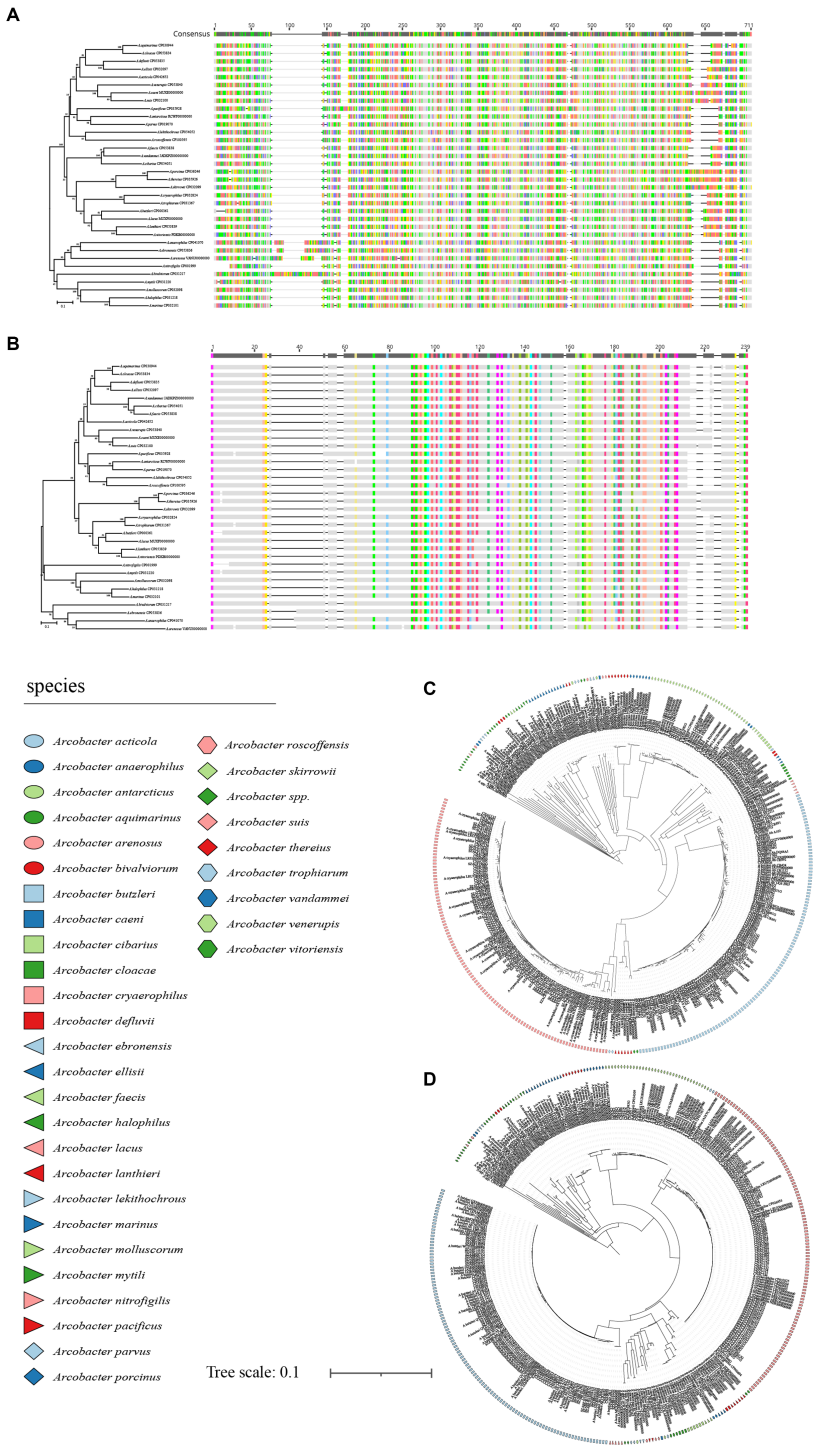
The NJ tree was constructed based on gene711, with a bootstrap value of 1,000. **(A)** was the phylogenetic tree constructed using the nucleotide sequence of 34 *Arcobacter* species type strain, **(B)** was the phylogenetic tree constructed using amino acid sequence of 34 *Arcobacter* species type strain, **(C)** was the phylogenetic tree constructed using the nucleotide sequence of 371 *Arcobacter* genomes, **(D)** was the phylogenetic tree constructed using amino acid sequence of 371 *Arcobacter* genomes. Different colors or shapes indicated different *Arcobacter* species.

The analysis of 371 genomes revealed a total of 835,009 genes 10,652 orthogroups, and 3,395 unassigned genes. Among these orthogroups, 216 were found to be present in all analyzed genomes, with 84 of them being single-copy orthologous genes. To elucidate the taxonomic relationships among members of the *Arcobacter* genus, we constructed a high-quality NJ phylogenomic tree based on the concatenation of these 84 conserved single-copy orthologous genes (Figure 4). The phylogenetic tree, derived from 84 single-copy homologous genes, demonstrated excellent resolution in identifying *Arcobacter* species. Notably, even *A. butzleri* and *A. lacus*, characterized by remarkably high ANI values, can be clearly differentiated. Remarkably, the species classification results derived from the phylogenetic tree using the 84 single-copy homologous genes closely aligned with the ANI results, which meant that *Arcobacter* can be accurately classified using single-copy concatenation genes. Phylogenetic trees for each single-copy orthologous gene were also constructed using nucleotide and amino acid sequences. When comparing the phylogenetic trees constructed based on nucleotide and amino acid sequences of each gene with ANI results, it was found that the topology of the phylogenetic tree built using gene711 was nearly identical to the phylogenetic tree constructed using the concatenation of 84 single-copy homologous genes (Figure 5; Supplementary Figure S2). During the sequence alignment analysis of each gene, gene711 effectively differentiated all species within the *Arcobacter* genus. Furthermore, the sequence similarities within species were found to be >96% (except for *A. cryaerophilus* and *A. marinus*), while the maximum sequence similarity between different species was <94%. Consequently, gene711 could be considered a reliable signature gene for identifying *Arcobacter* species, with a sequence similarity threshold of greater than 95–96% defining the same species (Table 2; Supplementary Tables S2, S3).

### *Arcobacter cloacae*, *Arcobacter lanthieri*, *Arcobacter skirrowii*, *Arcobacter marinus*, and *Arcobacter cryaerophilus* classification using the marker gene

**Figure 6:**
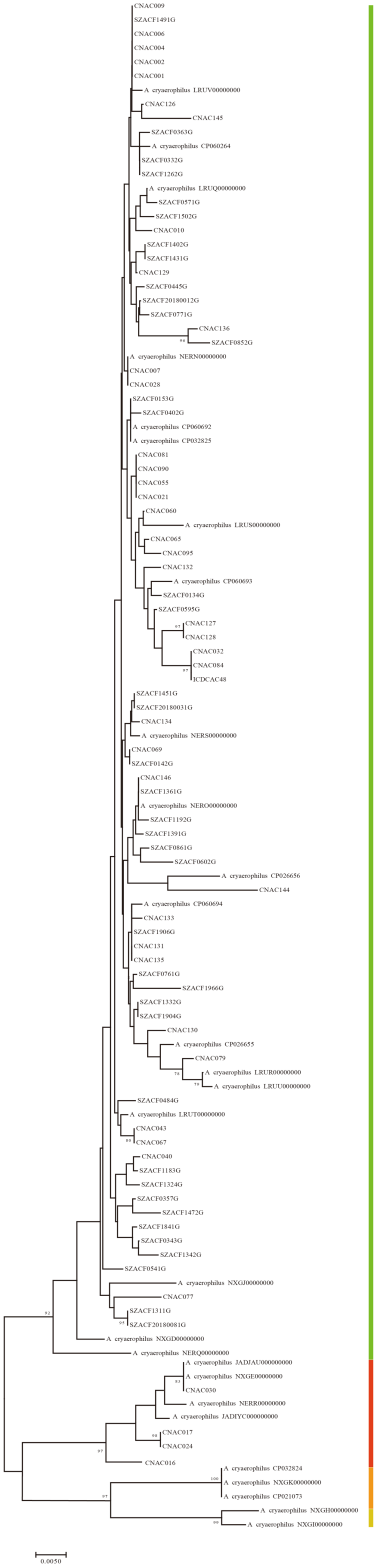
The phylogenetic tree was generated based on the sequences of gene711. The neighbor-joining method was used to generate the phylogenetic tree, which was performed using MEGA 7.0 with 1,000 bootstrap replications. Bars of different colors represented different subclades. Bar indicated 5 substitutions per 1,000 bp.

The gene711 exhibited sequence similarity above 96% in *A. cloacae*, *A. lanthieri*, and *A. skirrowii*, while within these species, their ANI and *isDDH* values were below the classification thresholds of 96 and 70%, respectively. In *A. marinus*, *A.marinus*_CP042812, *A. marinus*_NWVW00000000, and *A. marinus*_PTIW00000000 showed gene711 sequence similarities ranging between 95 and 96% with other genomes, which was consistent with the ANI and *isDDH* results. For *A. cryaerophilus*, except for CNAC091 and *A. cryaerophilus*_NERP00000000, gene711 effectively divided *A. cryaerophilus* into four distinct subspecies, as shown in Figures 3B, 6 and Supplementary Table S3. The sequence similarity of gene711 was >96% within each subspecies, while the sequence similarity between subspecies was <96%, similar to the results based on ANI and *isDDH*.

## Discussion

*Arcobacter* is recognized as a globally emerging foodborne and zoonotic pathogen with a wide range of sources and regions (Collado and Figueras, 2011; Ferreira et al., 2016). Understanding its genomic and classification characteristics is crucial for further investigations of this pathogen. In this study, a total of 371 genomes, comprising 34 named *Arcobacter* species and 14 unclassified *Arcobacter* species, were selected to elucidate the taxonomic characteristics of *Arcobacter*. The quality of the genome sequences generally met the minimal standards established for using genome data for taxonomical purposes (Chun et al., 2018). Globally, the genome size ranged from 1.68 Mb to 3.57 Mb. The G + C content ranged from 26.08 to 31.00%. Significant variations in genome size and GC content were observed in *Arcobacter*, suggesting considerable genomic diversity and divergence. This aspect could be one of the reasons contributing to the current challenges in the taxonomic classification of *Arcobacter*.

Like other bacterial genera, the taxonomic classification of *Arcobacter* has traditionally been based on the analysis of the 16S rRNA gene (Wesley et al., 1995). In fact, several potential new *Arcobacter* species could be inferred from the sequences available in public databases, similar to the 17 genomes downloaded in this study, which included 14 potentially new *Arcobacter* species. In previous studies, the similarity of the 16S rRNA gene has been considered a decisive characteristic for taxonomic classification at the genus or species level (Stackebrandt, 2006). Specifically, the sequence similarity of >98.7% in the 16S rRNA gene has been found to show good consistency with an *isDDH* > 70% (Stackebrandt, 2006). The sequence similarity of the 16S rRNA gene in 34 type strains of *Arcobacter* among multiple species was observed to be >98.7%. Moreover, expanding the number of 16S rRNA gene sequences to 281 revealed that more species displayed 16S rRNA gene sequence similarities >98.7%. However, it was necessary to note that phylogenetic trees constructed solely based on the 16S rRNA gene could cluster individuals of the same species together; however, relying solely on the 98.7% similarity threshold for species classification might lead to biased results. In other words, the discriminatory power of the 16S rRNA gene was limited when dealing with species that possessed highly similar 16S rRNA gene sequences. The 23S rRNA gene sequences were also attempted to assess *Arcobacter* interspecies differences, as published data indicated 16S rRNA gene sequences did not contain sufficient information to effectively discriminate between strains (Deshpande et al., 2013). However, our findings indicated that the 23S rRNA gene sequences were also insufficient for effective discrimination, likely due to the increased burden of additional sequences. Despite our efforts, the results obtained using the 23S rRNA gene were similar to those obtained using the 16S rRNA gene, further underscoring the limited discriminatory power for species with high sequence similarity.

Nowadays, genomic data such as the ANI and the *isDDH* are being increasingly used to define bacterial species, although their full potential for delineating genera has yet to be explored (Perez-Cataluna et al., 2018b; On et al., 2021). As discussed in other studies, the ANI and *isDDH* indices have been proven to provide reliable information for the delineation of *Arcobacter* species and have also been included in the minimal guidelines for defining species using genomes (Chun et al., 2018; Perez-Cataluna et al., 2018b; On et al., 2021). For *Arcobacter*, ANI values >96% were the ones that better correlated with *isDDH* results >70% in previous studies (Perez-Cataluna et al., 2018a; Zhou et al., 2022), which was further confirmed in this study. The ANI values between genomes of most *Arcobacter* species were consistent at >96%, except for certain genomes in *A. cloacae*, *A. lanthieri*, *A. marinus*, *A. skirrowii*, and *A. cryaerophilus* that did not meet the 96% classification threshold. Additionally, *isDDH* analysis was performed on species with ANI values <96%, and the results were consistent with the ANI result. Specifically, for genomes with ANI values<96%, their *isDDH* values were found to be <70%. For ANIm, intraspecies pairs generally have >96% identity, while interspecies pairs generally have <93%, with an intermediate range of 93–96% where species circumspection cannot be assured (Rossello-Mora and Amann, 2015). These findings suggested substantial genomic differences within *Arcobacter* species, even though they could be classified into different subspecies. Previous studies have proposed that *A. cryaerophilus* should be divided into four subspecies according to the species classification criteria of ANI values >96% and *isDDH* values >70% (Zhou et al., 2022), which was further confirmed in this study. Within the *Arcobacter* genus, 17 genomes potentially represented 14 new potentially species. The ANI values between these new species and the known genomes of *Arcobacter* exhibited significant differences. Only the ANI between *A.* spp._PDJV00000000 and *A. nitrofigilis*_CP001999 > 90% and reached 91.64%, while the ANI for the remaining genomes <90%. These findings further emphasized the substantial genomic diversity within the *Arcobacter* genus, which posed challenges for population classification.

This study established a method based on the construction of phylogenetic trees using single-copy orthologous genes for the rapid and simplified classification of *Arcobacter* species. A robust means of species identification within *Arcobacter* was provided by utilizing 84 single-copy orthologous genes. However, this method was not widely endorsed due to its reliance on a considerable number of genes. Fortunately, we have discovered that gene711 effectively differentiated various species within *Arcobacter*. The gene711, which encoded a 186–218 amino acid in *Arcobacter*, was a FlgO family outer membrane protein and was capable of reproducing a tree with a similar topology to our genome-based phylogeny. The gene711 sequences demonstrated high nucleotide diversity and yielded a tree that accurately separates strains into phylogenetic groups defined by ANI-based analysis. The gene711 exhibited sequence similarity >96% within the same species, while the similarity between different species was significantly <96%. The neighboring genes upstream and downstream of gene711 also displayed relatively conserved characteristics, making them potential targets for developing sequence-based analysis or real-time PCR assays to detect *Arcobacter* species. The discriminatory power of the gene711 locus made it possible to improve the accuracy of species identification within the *Arcobacter* genus. As mentioned earlier, certain genomes within *A. cloacae*, *A. lanthieri*, *A. marinus*, *A. skirrowii*, and *A. cryaerophilus* did not meet the species classification criteria of ANI values >96% and *isDDH* values >70% within the same species. Among these species, we used gene711 to verify and found that except for *A. marinus* and *A. cryaerophilus*, the remaining species met the requirement of gene711 > 96% within the species and gene711 < 96% between species. Previous studies (Zhou et al., 2022) have identified four subspecies within *A. cryaerophilus*, and our study using gene711 for *A. cryaerophilus* subspecies classification further supported this conclusion. However, there were also instances of gene711 anomalies in certain strains within *A. cryaerophilus*, such as CNAC091.

To our knowledge, this is the first time that gene711 has been used as a phylogenetic marker within a bacterial genus. As highlighted in the review by Collado and Figueras (2011), numerous uncultured or as-yet-undescribed species of *Arcobacter* have been identified based on nearly full-length 16S rRNA gene sequences, potentially surpassing the number of already known species at that time. The emergence of new species can be anticipated in the near future, further validating the significance of gene711 proposed in this study.

## Conclusion

In this study, we evaluated the efficacy of various genome-based phylogenetic tools in discriminating between different *Arcobacter* species. Novel approaches for the classification of the *Arcobacter* were employed in this study. Finally, a maker gene (gene711) that demonstrated greater discriminatory power and robustness than other commonly used markers was identified, making it a valuable tool for future molecular identification of *Arcobacter* species. In summary, our study offers valuable insights into the evolution, genetic diversity, and species classification of *Arcobacter*, thereby shedding new light on the behavior and characteristics of this genus.

## Data availability statement

The datasets presented in this study can be found in online repositories. The names of the repository/repositories and accession number(s) can be found in the article/Supplementary material.

## Author contributions

GZ: Methodology, Software, Writing – original draft, Writing – review & editing. YG: Writing – review & editing. HW: Software, Writing – review & editing. XC: Writing – review & editing. XZ: Writing – review & editing. ZS: Supervision, Writing – review & editing. XY: Supervision, Writing – review & editing. JZ: Supervision, Writing – review & editing. MZ: Methodology, Supervision, Writing – review & editing.

## Footnotes

^1^https://lpsn.dsmz.de/genus/arcobacter

^2^https://github.com/tseemann/barrnap

^3^Available at http://ggdc.dsmz.de/ggdc.php.

^4^https://github.com/dongwei1220/EasySpeciesTree

^5^https://itol.embl.de/
