# Supplementary figures and images for: Genomic diversity and taxonomic marker for Arcobacter species
Source: Front Microbiol. 2023 Oct 10;14:1278268. doi: 10.3389/fmicb.2023.1278268 (PMC10594997; doi:10.3389/fmicb.2023.1278268)

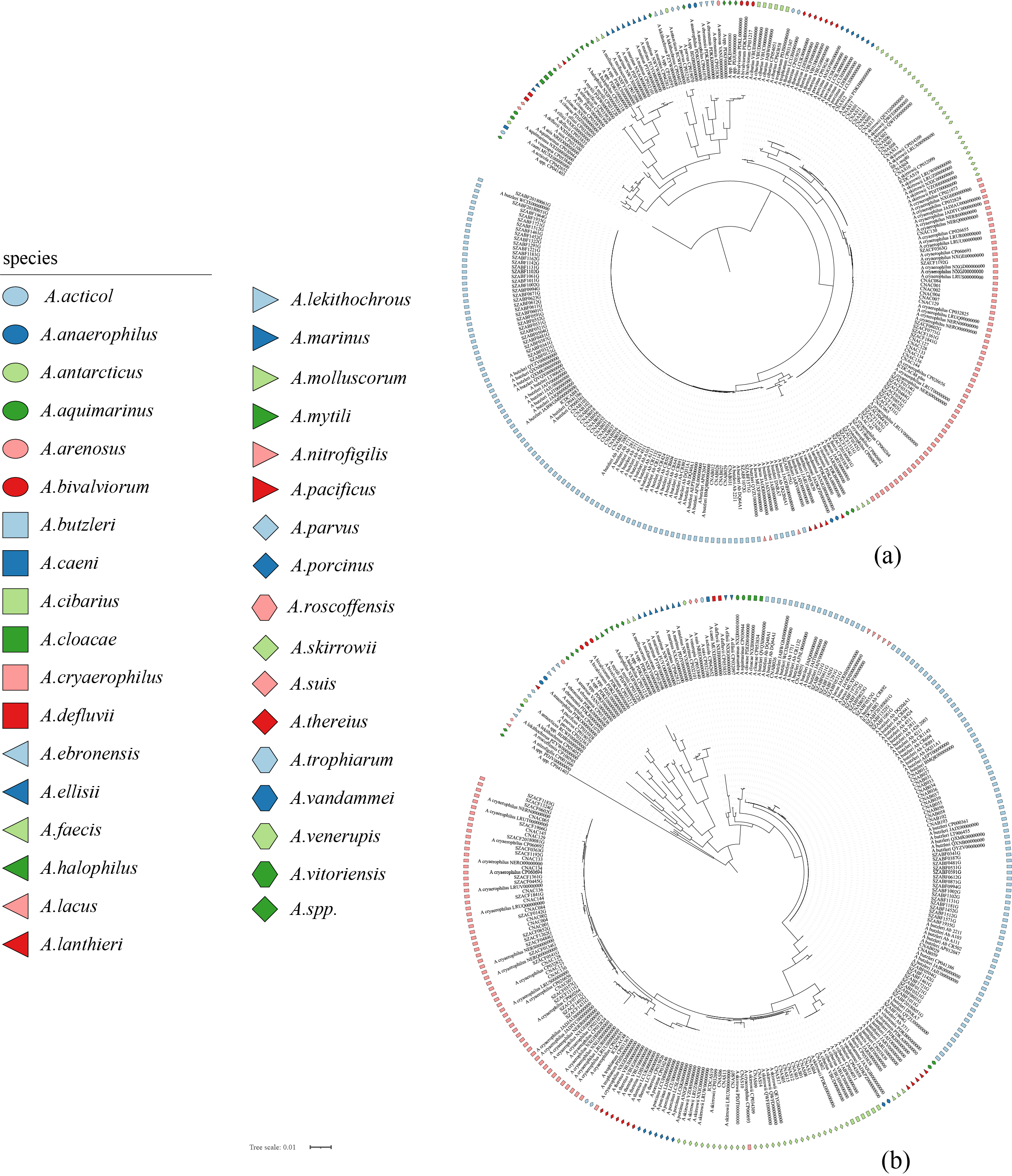

Supplement: Supplementary file 4 [file Image_1.TIF]

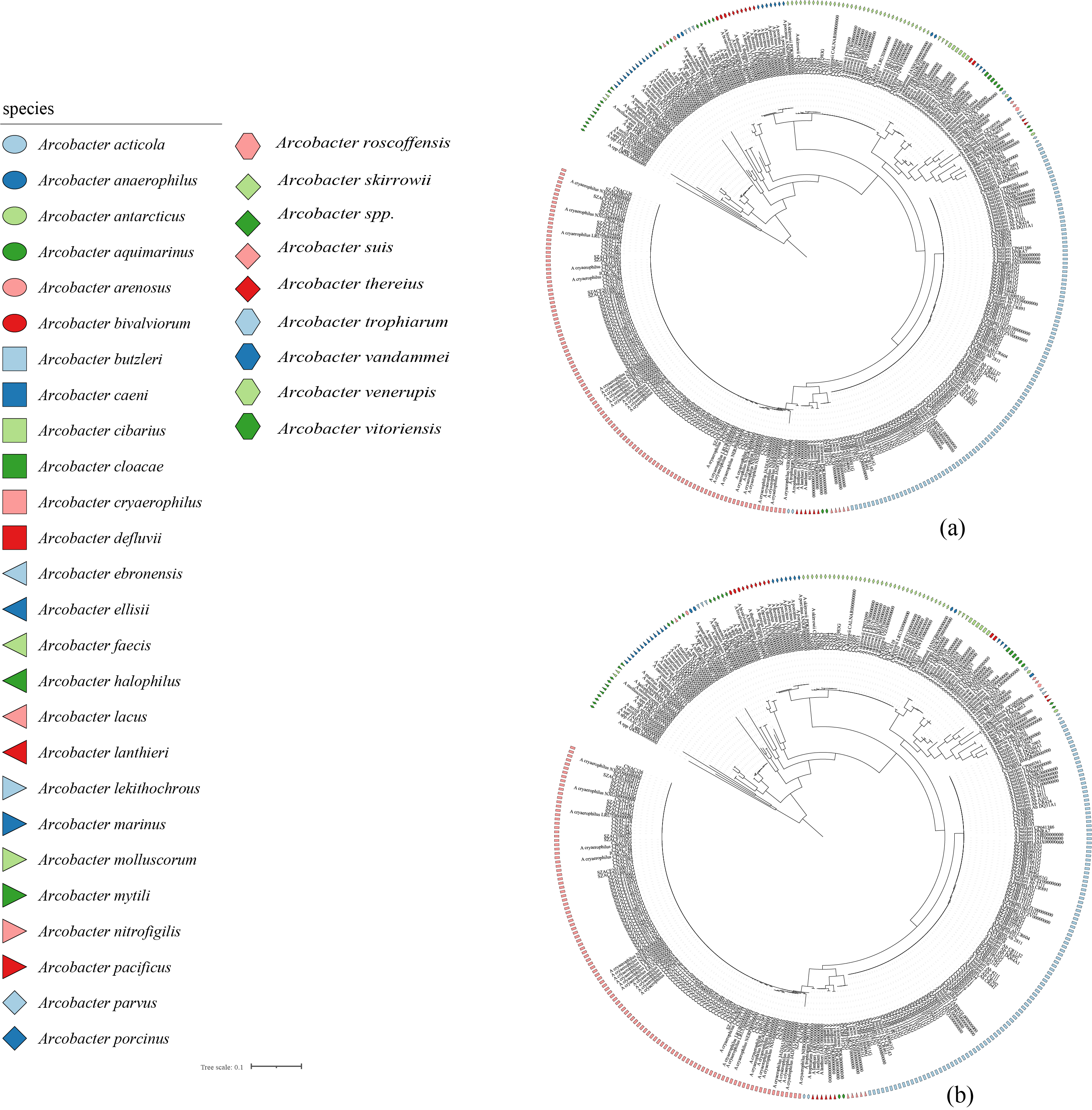

Supplement: Supplementary file 5 [file Image_2.TIF]
